# Effects of early home-based strength and sensory-motor training after total hip arthroplasty: study protocol for a multicenter randomized controlled trial

**DOI:** 10.1186/s13063-022-06779-8

**Published:** 2022-11-08

**Authors:** Pika Krištof Mirt, Vojko Strojnik, Gregor Kavčič, Rihard Trebše

**Affiliations:** 1 General Hospital Novo Mesto, Novo mesto, Slovenia; 2 Faculty of Medicine, University of Ljubljana, Ljubljana, Slovenia; 3 Faculty of Sport, University of Ljubljana, Ljubljana, Slovenia; 4 Valdoltra Orthopedic Hospital, Ankaran, Slovenia

**Keywords:** Total hip arthroplasty, Postoperative rehabilitation, Early postoperative exercise, Home-based training

## Abstract

**Background:**

Total hip arthroplasty (THA) is very effective in alleviating pain, but functional deficits persist up to a year following surgery. Regardless of standard physiotherapy programs, significant additional muscular atrophy and weakness occur. Deficits in strength have serious adverse consequences for these patients with respect to physical function, the maintenance of independence, and the requirement for revision surgery. Progressive resistance training in rehabilitation following THA has been shown to significantly enhance muscle strength and function. The fundamental principle is to progressively overload the exercised muscle as it becomes stronger. Different strength training protocols have been used at different times in the postoperative phase, in group or individual practices, with major differences being in center-based and home-based programs with or without supervision. The primary objective of our study is to evaluate whether an early postoperative home-based strength training protocol can improve patient functional outcomes at 3 months and 1 year following surgery. Secondary objectives are the feasibility of the presented protocol for all elective THA patients and its safety.

**Methods/design:**

This study is a prospective multicenter randomized clinical trial to be conducted in the orthopedic departments of two Slovenian hospitals. In each hospital, 124 patients aged 60 or older with unilateral osteoarthritis, an ASA score between 1 and 3, a signed informed consent form, and no terminal illness disabling rehabilitation participation will be randomly assigned to the intervention or control group. THA with an anterior approach will be performed. All patients will receive current standard physiotherapy during hospitalization. Patients in the intervention group will also learn strength and sensory-motor training exercises. Upon discharge, all will receive USB drives with exercise videos, written exercise instructions, and a training diary. Physiotherapists will perform the assessments (physical tests and the maximal voluntary isometric contraction assessment), and patients will fill out outcome assessment questionnaires (the Harris Hip Score and 36-Item Short Form Health Survey) at baseline and 1, 3, and 12 months after surgery.

**Discussion:**

The main purpose of our study is to design a new standardized rehabilitation protocol with videos that will be effective, safe, and accessible to all Slovenian THA patients.

**Trial registration:**

ClinicalTrials.gov NCT04061993. Registered on 07 November 2019. Protocol ID: PRT_PhD. Version 1.

**Supplementary Information:**

The online version contains supplementary material available at 10.1186/s13063-022-06779-8.

## Background

Total hip arthroplasty (THA) is one of the most widely performed and clinically successful surgical procedures, and the number of THA operations is rapidly increasing [1–4]. The number of THA procedures is expected to continue rising worldwide due to the wide range of indications for the procedure, and the prevalence of osteoarthritis (OA) is expected to increase as the population ages; accordingly, intentions to improve the mobility of elderly people are expected to increase [1–3, 5, 6]. THA is intended not only to relieve pain but also to restore hip biomechanics. The restoration of hip biomechanics leads to a minimal number of functional deficits, secures the longevity of the implant, and improves the quality of life, mobility, joint stability, and locomotion [2, 7]. THA is very effective in alleviating pain, but functional deficits persist up to a year following surgery [3]. Addressing these functional deficits is increasingly important, and postoperative center- and home-based programs have proven beneficial [5]. As technology and surgical techniques have improved, patient expectations from THA have also increased, including an early return to normal physical activities and a recovery of functional independence [8]. Early targeted rehabilitation has been shown to reduce hospital length of stay (LOS) without increasing the complication rates after THA [7, 9–11]. Utilization of the direct anterior approach, which is performed in the internervous and intermuscular plane, enables a fast recovery with little pain after surgery and does not require postoperative precautions [12]. With the implementation of optimal multimodal perioperative care to enhance recovery, the average hospital LOS has additionally reduced [13–17]. A reduction in the hospital LOS has increased the need for an efficient exercise program beyond the initial standard rehabilitation program completed during hospitalization [6]. A disadvantage of these programs is the need for patients to exercise under the supervision of professional staff at a hospital or rehabilitation center. These programs are expensive due to the high costs associated with staff supervision, treatment, and transportation between locations and are unfriendly because of the need for transportation [6, 7]. Although there are many studies that have tested different rehabilitation protocols against the “standard” practice, no explicit definition of standard practice exists. Standard physiotherapy rehabilitation programs may comprise hip joint mobilization, strengthening of adjacent muscles without external loading or with low-resistance weight, and gait training [1–3, 18]. However, it is well known that regardless of a patient’s adherence to standard physiotherapy rehabilitation programs, significant additional muscular atrophy and weakness occur in his or her affected limb, which is often in deficit compared to the healthy limb in patients with unilateral OA prior to surgery [1, 3, 7, 11]. Suetta et al. found 13% and 9% reductions in the cross-sectional area of the quadriceps on the operated side at 5 and 13 weeks after THA, respectively, following the completion of a standard program [11]. Deficits in strength have serious adverse consequences for THA patients with respect to physical function, the maintenance of independence, and the requirement for revision surgery. Leg strength deficits have been associated with poor gait symmetry, slow walking speeds, impaired stair-climbing and chair-rising abilities, limited access to public transportation, and an exacerbated risk of falling and loosening of the prosthesis [1, 2, 4, 7, 11, 19, 20]. In contrast, progressive resistance training (PRT) is an effective method for inducing muscle hypertrophy and increasing muscle strength and functional performance in healthy and clinical populations, including elderly individuals [21]. PRT in rehabilitation following THA has been shown to significantly enhance muscle strength and function, and PRT has been shown to be the main factor in achieving significant functional improvements in rehabilitation programs used after home- or center-based programs after THA [2, 5, 11, 22]. First, studies with strength training programs after THA were conducted with patients who underwent surgery after femoral neck fractures [18, 23]. The key points of these studies were that PRT is safe and effective in geriatric rehabilitation after hip surgery; the patients’ strength, functional performance, and emotional state improve by physical training; and long-term continuation of the programs may prevent detraining effects [23]. The fundamental principle of PRT is to progressively overload the exercised muscle as it becomes stronger [22, 24]. Evidence-based recommendations for resistance exercise to improve strength and power are as follows: an exercise frequency of 2–3 times per week, an exercise intensity of 60–70% 1-repetition maximum (1-RM) for novice to intermediate exercisers, 8–12 repetitions, 2–4 sets, a rest interval of 2–3 min between sets with ≥ 48 h between sessions, and a gradual progression [21, 22]. Studies in patients who underwent elective THA because of unilateral OA had small sample sizes, but they indicated that there are benefits of strength training protocols, and no major adverse effects were noticed [2, 8, 11, 19, 22, 25]. Different strength training protocols were used at different times in the postoperative phase, either in group or individual practices, with major differences being in center- and home-based programs with or without supervision. Some studies comparing supervised home- and center-based rehabilitation programs for THA patients have found greater improvements in function and quality of life for home-based patients than for center-based patients [7, 26, 27]. Studies of unsupervised home-based exercise programs beyond the immediate postoperative rehabilitation period have also reported increases in hip muscle strength, walking speed, and function with respect to those in the postoperative rehabilitation period [7, 20, 28]. Furthermore, a home-based rehabilitation program with supervision in the early postoperative period has been shown to be less expensive and more accessible for THA patients than a center-based program [26, 28, 29]. The main shortcomings of all published research are small samples, wide variations in exercise protocols that make it impossible to compare them with each other and cost ineffectiveness of the protocols developed. The implementation of the new rehabilitation protocol also depends on the financial situation of society and the organizational capacity of the health system. Therefore, for our country, we cannot directly transfer the protocol from existing studies. In an aging society, independent, self-reliant individuals after THA are an invaluable benefit of such enhanced protocol, both in terms of the burden on the individual family and in terms of improving society as a whole. With better organized home-based rehabilitation, we can also further shorten the LOS in hospitals, perform more THAs, and shorten the long surgery waiting list, which is a major problem in our country.

In our hospital, we implemented a rapid recovery protocol for all THA patients in 2011. We continuously made changes, including improvements in the preoperative preparation of patients and their relatives with a multidisciplinary preoperative education program and the implementation of the direct anterior approach, shortening the patients’ hospital LOS from an average of 10 to 3 days and improving patient satisfaction. Inherently, the primary goal is safe mobilization, not functional optimization. To date, only minor changes have been made to the standard physiotherapy protocol, and there is still much room for improvement. Currently, patients receive written and pictorial instructions for standard home-based exercises. This existing standard physiotherapy protocol, which has been shown to be safe but probably not maximally effective, will serve as a comparator in the present study.

### Objectives

The primary objective of our study is to evaluate the efficacy of an early postoperative home-based strength training protocol — to assess whether it can improve patient functional outcomes at 3 months and 1 year following surgery. The secondary objectives are the feasibility of the presented protocol for all elective THA patients and its safety.

Our study also aims to assess patient satisfaction with enhanced targeted physiotherapy and to prepare a new standardized rehabilitation protocol with videos that will be effective, safe, and accessible to all THA patients.

## Methods/design

### Study design

**Fig. 1:**
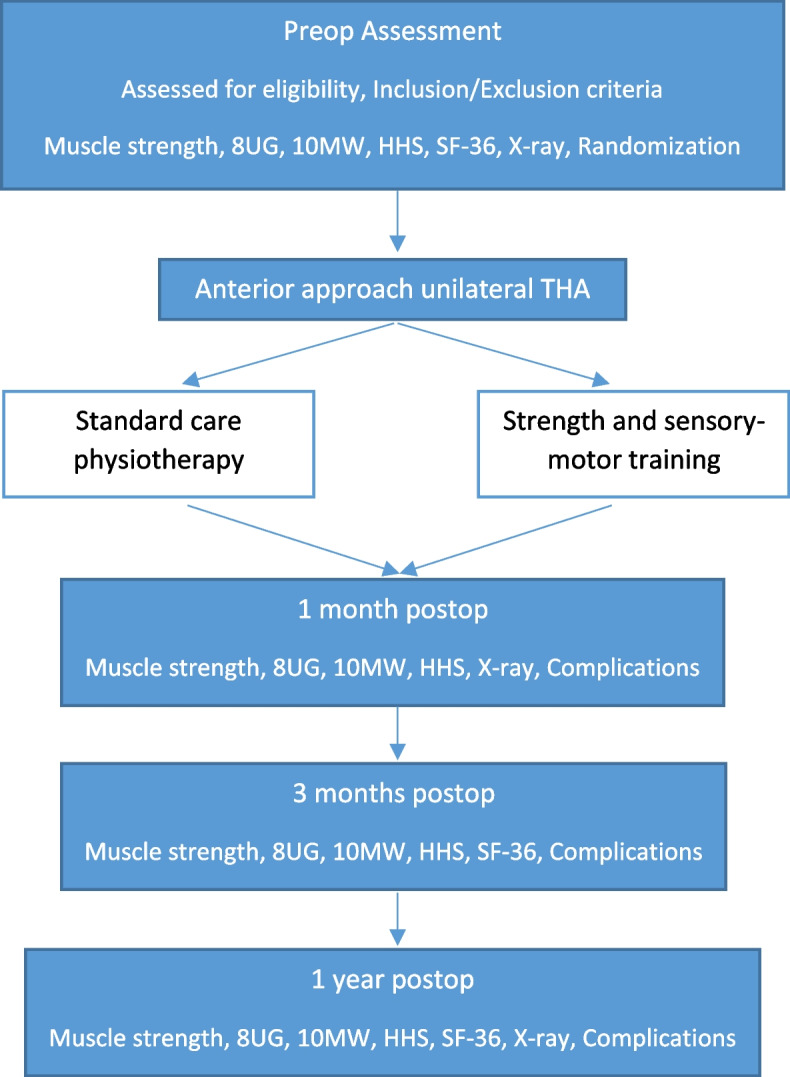
Trial flowchart

The study is a multicenter randomized controlled trial comparing the effect of intensive early postoperative physiotherapy with the current standard of physiotherapy for patients undergoing elective THA for unilateral OA. General Hospital Novo mesto and Valdoltra Orthopedic Hospital are involved in the project, and the standard physiotherapy program for patients after THA is similar in both hospitals. All patients who will receive an anesthesiologist’s approval and will already be scheduled for surgery will be assessed for eligibility. An orthopedic surgeon will invite patients to participate and give oral and written explanations about the trial. Patients will be included in the study after signing a written informed consent form. After admission to the hospital, they will complete the routine preoperative outcome assessment questionnaires (Harris Hip Score (HHS) and 36-Item Short Form Health Survey (SF-36)) and have standard preoperative AP X-rays of both hips taken. Physiotherapists will perform the assessments: physical tests and an isometric muscle strength assessment. In both hospitals, the included patients will be randomized to either the intervention group (IG) or the control group (CG), and the surgeons and patients will be blinded to the randomization process. The procedure will be performed with the direct anterior approach only in the General Hospital Novo mesto by 1 surgeon and in the Valdoltra Orthopedic Hospital by 4 surgeons. Patients will be mobilized on the day of surgery. During the expected LOS of 2–5 days, all patients will receive current standard physiotherapy, oral analgesics, and cryotherapy. Patients in the IG will receive extra one-on-one training to learn strength and sensory-motor exercises. If the surgeon applies any limitations for a patient regarding rehabilitation after surgery, the patient will be excluded from the study. At discharge, patients in both groups will receive USB drives with exercise videos, written exercise instructions, and a training diary. Patients in the IG will also receive exercise aids for the strength and sensory-motor training. All patients will be followed up with regular phone calls concerning possible complications, completion of the training diary, and the clarity of the exercises. The patients will visit the hospital as usual after 1, 3, and 12 months following surgery. At each follow-up, the physiotherapists will perform assessments, and patients will fill out questionnaires. The patients will have a standard postoperative X-ray of the operated hip immediately after the surgery and AP X-rays of both hips after 1 year, so we will be able to compare measurements of the hip offsets of the operated and healthy hips and observe any changes regarding component position or osteolysis. At every follow-up, the surgeon and physiotherapist will ask the patient about any adverse effects or reasons for training cessation. The trial flowchart is displayed in Fig. 1.

This paper is written according to the Standard Protocol Items: Recommendations for Interventional Trials (SPIRIT) 2013 Statement for the reporting of clinical trial protocols (Table 1, Additional file 1) [30, 31].

**Table 1:**
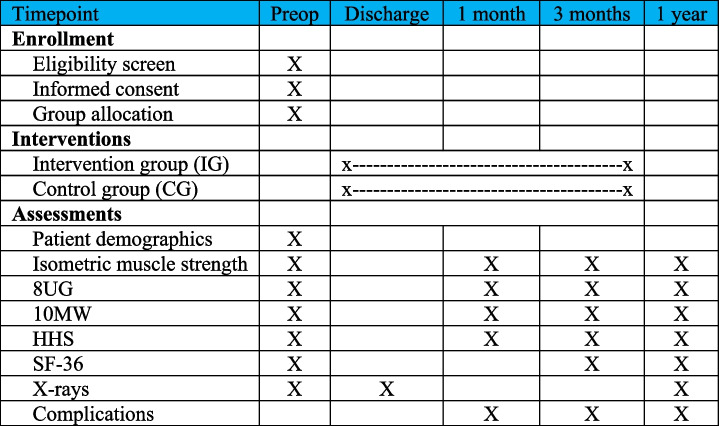
SPIRIT figure [30, 31]: schedule and outcome measurements preoperatively and at 1, 3, and 12 months postoperatively

### Study population

#### Inclusion criteria

1. Patients undergoing elective primary unilateral THA for OA
2. Patients older than 60 years at the time of surgery
3. Patients with the ability to watch exercise videos on a USB drive
4. Patients with an American Society of Anesthesiologists (ASA) classification 1–3

#### Exclusion criteria

1. Patients undergoing primary THA for a diagnosis other than OA (aseptic femoral head necrosis, dysplastic hip, or others) or revision THA
2. Patients with previous hip interventions (osteosynthesis, osteotomy…)
3. Patients discharged to rehabilitation units or nursing homes
4. Patients unable to consent and comply with the study protocol (diagnosed with dementia, mental disorders, poor preoperative physical status, neurological disorders, amputations, trouble walking with walkers or wheelchairs, or a painful hip or knee prosthesis in other joints).

#### Randomization procedures

Randomization will be performed in each hospital separately. Randomization will be made using a bespoke web-based randomization protocol. Patients will be randomized on a 1:1 ratio to intervention group A (with strength and sensory-motor training included) or control group B (with standard-of-care physiotherapy), regarding inclusion order. Physiotherapists and coordinators of the trial will be aware of the randomization procedure. Patients and orthopedic surgeons will be blinded, but they will be unblinded, if any safety issue or adverse effect arises.

#### Sample size calculation

A power calculation was performed based on a clinically significant difference in gait speed of 0.10 m/s on the 4- or 10-m walk (10 MW) test using data from Perera et al. [32]. Based on a substantial meaningful change of 0.10 m/s between the interventional and control groups and standard deviation of 0.28 m/s, with a significance level of 5% and a power of 80%, a needed sample size of 124 patients in each group was estimated (248 patients in total). All patients from surgeons, participating in the study, will be assessed for eligibility. A total of 124 patients in each hospital will be randomized according to the procedure described above on a 1:1 ratio, so that there will be 62 patients in group A and group B in both hospitals.

### Study intervention

#### Standard rehabilitation

All patients will receive standard-of-care physiotherapy during hospitalization. Physiotherapy will commence on the day of surgery with mobilization using a walking aid (usually two crutches, rarely a walker), deep vein thrombosis prevention exercises, lower limb range of motion exercises, and an isometric strengthening program. Patients will be encouraged to perform exercises twice daily, with approximately 10 repetitions of every exercise, and walk as much as possible. Exercises will be performed in a supine position, on the healthy side, on the abdomen, sitting and standing (e.g., buttock squeezes, leg sliding motions, straight leg raises, bridges, postural exercises). Patients will be discharged based on a combination of compliance with the exercises, stability with a walking aid, independence with activities of daily living, and dry wounds. Patients in the CG will receive written instructions, a USB drive with videos of standard exercises, and a training diary at discharge.

#### Prescribed home-based strength and sensory-motor training

Before the patients are discharged, physiotherapists will teach patients in the IG special strength and sensory-motor exercises with exercise aids. The patients will also receive USB drives with special exercise videos, written exercise instructions, and a training diary to check performed exercises. The new training protocol includes exercises for improving hip stability and reducing local stresses on the hip prosthesis. In the training, we considered safety regarding hip and lumbar spine load, falls, and prosthesis luxation. Training will consist of hip muscle strengthening exercises (focusing on the abductors), hip and pelvic stabilization exercises, ankle and knee muscle strengthening exercises (for improved dissipation of impact forces and femoral inner rotation control), and trunk muscle strengthening exercises (for stabilization of the pelvis and lumbar spine and a reduction in local loads). We ensured the exercises are simple and easy to understand, the videos that are to be watched at home are easy to follow, and the training program does not require expensive equipment. In the first two postoperative weeks, the focus is on learning the proper exercise technique and developing a sense of loading. Patients will watch special instructional short videos with exercise descriptions. Each exercise will be demonstrated with easy and challenging options as well as oral and written instructions. We will use a revised Borg category-ratio scale (0 to 10 scale) for perceived exertion to monitor and guide exercise intensity [33]. In sets of fluent concentric repetitions of movements (10–12 repetitions per set), the rating of perceived exertion will be recorded for the last repetition. When holding an isometric position, the rating will be recorded for the end of the interval (with a lengthening of the interval to 30 s, until we can achieve desired perceived exertion at the end of the interval). In sets of isometric contractions, every repetition will be made with the maximal voluntary effort exerted for 6 s (2 s for force increments and 4 s for maintenance). The implementation of fluent concentric movements is intended to increase muscle mass and improve physical endurance, while maximal isometric contractions are used for improving the level of muscular activation [34]. Exercises with isometric trunk positions will be prepared with recommendations for isometric trunk stabilization training [35]. These concepts collectively allow for individual load adaption for every exercise regarding individual patient abilities and load progression in accordance with his or her progress in muscle strengthening training [34]. Each session consists of a warm-up with 2 options (5–7 min of toe walking on the same spot or step-ups on a 10-cm tall step), strength training for both legs (30–40 min; 11 exercises; 1–4 fluent concentric repetitions of the single-leg squat and lying side hip raise; 5–8 maximal isometric contractions of the sitting ball squeeze, seated elastic band hip abduction, and diagonal ball push into the opposite thigh; 9–11 holds in the isometric positions, side plank, and bridge), and stretching (standing hip flexor stretch, sitting forward bend stretch, standing lateral trunk stretch). Strength training will be performed twice weekly, with a minimum of 2 days of rest. Until the 5th postoperative week, perceived exertion will be increased on the Borg scale from 5 to 8, and then it will be increased to 9 until the assessment at 3 months. In the first 5 postoperative weeks, 2 sets of each exercise will be recommended, and after these 5 weeks, 3 sets will be recommended. Sensory-motor training will be added to strength training to increase muscular activation and rate of force development and will be practiced on the days without strength training [36]. Exercise for ankle stability will be performed with a one-dimensional wooden balance board in the frontal and sagittal plane [37]. At the beginning, patients will perform 6 sets for each leg and 12 sets after the 7th postoperative week. The intensity of the exercise will be increased according to patient abilities with changes in the stability of the balance board. For every exercise, severe pain will be a reason for training cessation and contact with the physiotherapist.

### Outcome measures

The primary outcome of the study is the efficacy of the chosen protocol, measured by physical tests. These comprise the 10 MW test, the 8-foot Up and Go (8UG) test, and isometric muscle strength. Secondary outcomes are the patient questionnaires, which will be used to draw conclusions about patient satisfaction and the feasibility of the protocols, and consistent recording of any adverse effects.

#### Maximal voluntary isometric contractions

We use a specially prepared measuring device with a dynamometer attached to stiff band metal chains, prepared similarly to those in Roussel et al. and Essendrop et al. [38, 39]. The ICC for a similar device, used by one of the senior authors for lumbar muscle strength assessment, is > 0.94. For the assessment of maximal isometric strength, a maximal voluntary isometric contraction for 6 s is used. The same procedure of obtaining a maximal voluntary contraction is used for every strength measure. Movement is assessed in 3 planes for the trunk (extension, left and right abduction) and operated hip (flexion, extension, and abduction), and movement is assessed in 2 planes for the contralateral hip (extension, abduction). All strength tests are performed in a neutral standing position, and the patient rotates on the measuring device so that the exerted force is always in the direction opposite of the dynamometer. Before the measurements, the height of pelvic support is set to the reference point of the iliac crest of each patient. Patients are verbally encouraged to generate maximal effort. The mean duration of the complete test is 20 min. We wrap the band around the trunk under the armpits for trunk tests and around the ankle for leg tests. We measure the height of pelvic support, maximal contraction force, and distance between the upper border of the support board and the middle point of the stiff band. All measurements are taken twice.

#### Questionnaires

##### Harris Hip Score

The HHS is a widely used disease-specific measure of hip disabilities after THA. The physiotherapist administers the test in the form of a structured interview with the patients. The domains include pain, functions of daily living, and gait. The rating scale is from 0 (worse) to 100 points (best) [40]. The HHS is considered to have good validity and reliability [41].

##### 36-Item Short Form Health Survey

The SF-36 Health Survey was first made available in standard form in 1990. The eight health domains represented in the profile were selected from the 40 domains that were included in the Medical Outcome Study (MOS) by Stewart and Ware [42]. The RAND 36-Item Health Survey (version 1.0) is a set of generic, coherent, and easily administered quality-of-life measures. It taps eight health concepts: physical functioning, bodily pain, role limitations due to physical health problems, role limitations due to personal or emotional problems, emotional well-being, social functioning, energy/fatigue, and general health perceptions. It also includes a single item that provides an indication of perceived change in health [43]. These 36 items, presented in RAND 36-Item Health Survey 1.0, are identical to the MOS SF-36 described in Ware and Sherbourne [44]. Scoring of the RAND 36-Item Health Survey 1.0 is clearly described in scoring instructions [43]. The survey is a practical, reliable, and valid measure of physical and mental health. In our study, we will use the Slovenian translated and validated version by Marn-Vukadinović et al. [45].

#### Training compliance

All participants in both groups will keep a training diary concerning all training activities; they will make marks by every exercise they perform on each day, write notes if they will perform any other sport activities (walking, stationary bicycling, and others), note any adverse effects, and note reasons for skipped training exercises if applicable. They will also record their pain level by using the visual analog scale (VAS) [46] during the day, before and after training, and during the night. All these information will be used to draw conclusions about patient satisfaction, feasibility, and safety of the protocols.

### Data management and analysis

All data will be collected and stored in paper form by the corresponding author, who is responsible for the confidentiality of the data. All check-up forms will be printed and completed preoperatively and at every check-up by the physiotherapist and orthopedic surgeon. The questionnaires will also be printed and completed by patients. The corresponding author will enter the data into anonymized tables, which will then be used for statistical processing. The software program SPSS 21 (IBM SPSS Statistics for Windows, version 21.0. Armonk, NY: IBM Corp.) will be used for statistical analysis.

We will perform an intention-to-treat (ITT) analysis, which is an analytic strategy for reducing potential bias in treatment effects arising from missing data in randomized controlled trials. In ITT analysis, a study participant is analyzed as belonging to whatever treatment group he/she was randomized into, whether or not the treatment course was completed as intended. In case of missing data, sensitivity analyses will be performed and reported.

Before any statistical analysis, data will be tested for normal distribution using the Kolmogorov–Smirnov test (K–S test). With normally distributed variables, the analysis of variance for repeated measures (AnovaRM) with two factors (time × group) will be employed. It will test the differences in the effects of two different exercise protocols. AnovaRM will be used to analyze between-group differences in the score changes among all time points, represented by preoperative measurements (on admission to the hospital) and measurements 1, 3, and 12 months postoperatively. Afterwards, the initial AnovaRM, post hoc tests with AnovaRM (time × group) with two time points will be performed. Differences between all pairs of time points will be analyzed. Additionally, the paired samples *t*-test to test for in-group differences in the score changes between the pairs of time points and the independent samples *t*-test for between-group comparisons at the same time point will be calculated. The bivariate Pearson correlation will be calculated for testing the relationships between variables representing relative changes in the results between two different time points (e.g., post-treatment/baseline) for each group separately. To test the reliability of maximal voluntary isometric contraction measurements and the 8UG test, we will use the intraclass correlation coefficient (ICC) with the two-way mixed-effects model with absolute agreement. Data will be presented as the arithmetic mean and standard deviation. In the case of a non-normal distribution, data will be presented as the median and interquartile range, and nonparametric tests will be used respectively for comparisons of dependent and independent groups. Instead of AnovaRM, the nonparametric marginal model (nparLD, R-statistics) [47] will be used. T-tests for dependent and independent samples will be changed with Wilcoxon matched-pair signed-rank test and Mann–Whitney *U* test, respectively. The Pearson correlation will be substituted with the Spearman rank correlation. Adverse effects comparison among the two groups will be made by using the chi-square test. We will consider a *P* value less than 0.05 as significant for all measures and use a 95% confidence interval (CI). A significance level (alpha) of 0.05 will be tested with a two-tailed test.

### Data monitoring

Instead of a data monitoring committee (DMC), the presented study is supervised by a doctoral thesis review committee. It is composed of 3 members, whose names are available on request. The corresponding author is obliged to present the study design to the panel before starting the enrollment and to present the interim results and finally conclusions of the study. An interim analysis will be made after the completion of the patient recruitment, at which time the results will also be presented to the panel and any adverse effects recorded will be discussed. Given the existing data from the previous studies, we do not expect any major side effects requiring discontinuation of the study, except that it may be necessary to discontinue the protocol in individual patients.

## Discussion

We have had good experiences with the standard exercise protocol, so we anticipate that the majority of the patients from both groups will have better performance at 3 months and 1 year after surgery compared to at baseline. All patients should also have higher scores on patient satisfaction questionnaires at 3 months and 1 year after surgery than at baseline. We expect patients in the intervention group to achieve better results in physical tests and maximal voluntary isometric contraction measurements compared to patients in the control group. With respect to other studies, we predict the largest differences at the 3rd postoperative month. We would like to provide evidence that home-based strength and sensory-motor training is safe and easy to understand for the majority of patients and is applicable to all THA patients.

### Future directions

The aim of our project is to design a safe, affordable home-based strength and sensory-motor training program for all THA patients that can integrate well with the existing rapid recovery protocol in our hospitals.

## Trial status

Patient recruitment for our study commenced in January 2018 in the General Hospital Novo mesto and in April 2018 in the Valdoltra Orthopedic Hospital and is on-going at the time of the manuscript submission. The expected time of recruitment completion was June 2021, but was postponed due to COVID-19.

## Supplementary Information

**Additional file 1.** Standard Protocol Items: Recommendations for Interventional Trials (SPIRIT) 2013 Checklist: Recommended items to address in a clinical trial protocol and related documents.

## Data Availability

The datasets used and analyzed during the current study are available from the corresponding author upon reasonable request.

## Abbreviations

THA: Total hip arthroplasty
PRT: Progressive resistance training
IG: Intervention group
CG: Control group
HHS: Harris Hip Score
SF-36: 36-Item Short Form Health Survey
OA: Osteoarthritis
LOS: Length of stay
1-RM: 1-Repetition maximum
10MW: 10-M walk
8UG: 8-Foot Up and Go
TUG: Timed Up and Go
ASA: American Society of Anesthesiologists
VAS: Visual analog scale
ICC: Intraclass correlation coefficient
